# Legalizing Cannabis: A physician’s primer on the pulmonary effects of marijuana

**DOI:** 10.1007/s13665-014-0093-1

**Published:** 2014-10-12

**Authors:** Denyse Lutchmansingh, Leena Pawar, Dana Savici

**Affiliations:** Division of Pulmonary and Critical Care Medicine, SUNY Upstate Medical University, 750 East Adams Street, Syracuse, NY 13210 USA

**Keywords:** Habitual smoking, Marijuana, THC, Respiratory care

## Abstract

Habitual smoking of marijuana is associated with multiple respiratory symptoms such as cough, sputum production, and wheezing .These symptoms are not significantly different from those exhibited by tobacco smokers. Furthermore, endobronchial biopsies of habitual smokers of marijuana and /or tobacco have shown that both marijuana and cigarette smoking cause significant bronchial mucosal histopathology and that these effects are additive. Although marijuana smokers have minimal changes in pulmonary function studies as compared to tobacco smokers, they may develop bullous disease and spontaneous pneumothoraces. The relationship between marijuana smoking and lung cancer remains unclear due to design limitations of the studies published so far. These findings should warn individuals that marijuana smoking may result in serious short-term and long-term respiratory complications, and habitual marijuana use should be viewed with caution. The medical literature so far does not support routine evaluation by pulmonary function tests or imaging studies; until more definitive data is available, we do not recommend the regular use of these tests in the evaluation of habitual marijuana smokers.

## Introduction

In 2012, an estimated 23.9 million Americans aged 12 or older were current illicit drug users. Marijuana (cannabis) was the most commonly used drug, with 18.9 million users, an increase from 14.5 million between 2007 and 2012 [1]. As drug-related laws evolved with the enactment of Colorado’s Amendment 64 and the similar Washington Initiative 502, the legalization of marijuana has increased accessibility to a drug whose principle route of administration is via inhalation. Therefore, knowledge of the pulmonary complications of marijuana use is relevant to current medical practice.

Historically marijuana use dates back as far as 2700 BC China with a variety of roles ranging from textiles to medicinal use [2]. Medical indications for cannabis included rheumatic pain, intestinal constipation, disorders of the female reproductive system, malaria, and others [3] . The medical use of cannabis spread from Europe to the Americas much later with culmination of use in the second half of the 19th century [2] . By this time over 100 articles had been published on the therapeutic indications of marijuana [4] with different laboratories including Merck (Germany), Burroughs-Wellcome (England), Bristol-Meyers Squibb (United States), Parke-Davis (United States), and Eli Lilly (United States) marketing *tincture of cannabis* [5] . However there was a decline in its use in medical practice in the 20th century and it was removed from the American pharmacopoeia in 1941[6].

Marijuana is derived from the Cannabis sativa plant and the active ingredients known as “cannabinoids” are found within the stalks, leaves, flowers, and seeds of the plant [7]. The drug is prepared using the dried flower tops and leaves. The major psychoactive agent in marijuana is 1-δelta-9-tetra hydrocannabinol (THC) with additional cannabinoid-like compounds and tar components similar to tobacco smoke [8]. However, more than 400 compounds have been identified in marijuana, including 60 different cannabinoids [9]. These include cannabinol (CBN), cannabidiol (CBD), and delta-8-tetra hydrocannabinol, which do not produce significant psychoactive effects [7]. The average cannabinoid content in dried marijuana leaves in the United States between 1993 and 2008 was 4.5 % THC, 0.4 % CBD, and 0.3 % CBN [10]. Hashish, which is a more concentrated form, was noted to have a higher THC concentration (14.1 % THC, 2.4 % CBD, and 1.9 % CBN) [10]. The THC content and potency of marijuana joints have increased when compared to the 1960s and 1970s. In the 1960s the average THC content of a joint was 10 mg [9]. In current rolled cigarettes this has increased to 150 mg and up to 300 mg if hashish oil is added [9]. Potency of THC in marijuana samples has steadily increased from 3 % in 1980 to 12 % in 2012, raising the concern that the consequences of marijuana use may be worse now than reported in past literature.

## Marijuana and the airways

Several reports in the last few years have indicated that inhaled marijuana may, in fact, dilate the airways of healthy and asthmatic individuals, an effect that lasts only a few minutes to hours. However, THC, which is known to act as a short-term bronchodilator, also has long-term biological effects. These effects may cause structural and functional changes in the lung parenchyma and airways.

Habitual marijuana smokers report a wide range of symptoms such as cough, dyspnea, sputum production, wheezing, pharyngitis, hoarseness of voice, and worsening of asthma symptoms [11] . The symptoms are comparable to the symptoms exhibited by tobacco smokers who have smoked for at least 10 years. Tashkin et al. assessed the respiratory function in seventy-four habitual marijuana smokers and a matched group of controls. Fifty of the seventy-four subjects had never smoked tobacco or had not smoked tobacco for 6 months. The researchers noted that among marijuana smokers and/or tobacco users, the prevalence of chronic cough was 18 to 24 %; sputum production was 20 to 26 %, and wheeze was 25 to 37 %, with at least two prolonged episodes of acute bronchitis during the previous 3 years (10–14 %), significantly higher than among the non-smokers (*p* <0.05). Nonetheless, there was no difference in the prevalence of chronic cough, sputum production, or wheeze between the marijuana and tobacco smokers or combined smokers of both marijuana and tobacco [12].

Spirometric evaluation of lung function demonstrated an increased airflow resistance in young habitual smokers of marijuana [12] . Furthermore, a study by Aldington et al. included subjects who smoked both marijuana and tobacco in addition to those smoking marijuana or tobacco alone[13]. Nonsmokers represented the control group. 339 patients returned a respiratory and smoking questionnaire and underwent pulmonary function testing in addition to HRCT. The authors found a dose–response relationship between cannabis smoking and decreased FEV1/FVC ratio, increased specific airway conductance, and increased total lung capacity. The lung density on HRCT was decreased with macroscopic emphysema in 16.3 % of the combined group and 1.3 % in the cannabis-only group.

The results of the study by Tashkin raised the possibility of histologic changes in the mucosa of the central airways which might be qualitatively similar to the changes present in the mucosa of tobacco smokers [12]. Airway histology was studied by Fligiel et al. in habitual smokers of cocaine, marijuana, and tobacco by using bronchoscopy and endobronchial biopsies [14]. The authors found that both smokers of marijuana and cigarettes had significant bronchial mucosal abnormalities and that these effects were additive. They reported that 20–26 % of young smokers of marijuana and/or tobacco displayed some symptoms of chronic bronchitis and as many as 80 % had evidence of cellular atypia and mucosal metaplasia in the major bronchi.

The same group also assessed early effects of marijuana and tobacco smoking on the airway mucosa using a visual bronchitis index assessed by bronchoscopy and transbronchial biopsies [15]. The bronchitis index was defined as vascular proliferation, edema, cellular infiltrates, and goblet cell hyperplasia. The average screening spirometry was normal in all subjects. Both marijuana and tobacco smoking were associated with visual evidence of central airway inflammation in the majority of subjects. All biopsies from subjects smoking marijuana, tobacco, or both were graded as abnormal. Mucosal erythema was present in 95 % of samples, with three of the four bronchial index criteria present in 72 % of subjects and 48 % of subjects fulfilling all four. The IL-8 concentration in the BAL correlated with the BAL neutrophil counts, and both were significantly increased in smokers of both marijuana as well as tobacco.

A cohort study by Hancox et al. evaluated the lung function in 1,037 individuals with 71.6 % of subjects having smoked marijuana at least once in their lifetime [16]. In this study cumulative marijuana use was associated with higher forced vital capacity, total lung capacity, functional residual capacity, residual volume, and airway resistance, but there was no significant change in the forced expiratory volume in 1 s, or diffusing capacity. Tobacco smokers on the other hand, had higher lung volumes suggestive of hyperinflation and increased large airways resistance.

## Marijuana and bullous lung disease

Although marijuana has not been linked to the development of chronic obstructive lung disease, asymmetrical bullous disease in the setting of normal chest X rays and lung function has been described. Johnson et al. [17] published a small case series of four patients with significant exposure to cannabis and limited tobacco use. They noted a form of bullous disease with paraseptal distribution and a marked predisposition for the upper lobes with little other parenchymal lung disease in young male cannabis smokers. Hii et al. [18] reported similar findings in a series of ten patients with new respiratory symptoms and one or more years of marijuana use. High-resolution CT of the chest demonstrated variably sized, asymmetrical emphysematous bullae in the upper and mid-lung zones.

Fiorelli et al. [19] have pointed to marijuana as a risk factor for the development of bullous disease and pneumothorax. His group has retrospectively evaluated 13 consecutive habitual marijuana smokers who were referred for treatment of spontaneous pneumothorax. In patients less than 35 years of age, the incidence of bullae was higher in marijuana smokers, defined as a lifetime total of at least 50 marijuana cigarettes, compared to non marijuana smokers (7/10 vs. 3/10 patients, respectively, *p* < 0.05). Radiological findings on CT chest were similar to those reported in the prior studies with upper lobe-predominant paraseptal distribution of bullae. Chest x-rays, however, showed no evidence of emphysema.

Furthermore, Beshay et al. [20] presented a retrospective case series of 17 patients less than 40 years of age who presented with spontaneous pneumothoraces and significant upper lobe-predominant bullous emphysema. All but one subject smoked marijuana daily for a mean of 8.8 years and tobacco for 11.8 years. This has led to the term “marijuana lung “or “bong lung” where patients typically develop large peripheral paraseptal lung bullae and are predisposed to spontaneous pneumothoraces [21].

The exact mechanism of bulla formation in marijuana smokers is not well understood. However it has been suggested that development of bullous disease and subsequent pneumothorax is related to inhalation techniques of marijuana resulting in barotrauma. Common techniques involve deep inhalation to hold smoke in the lung, performing a Valsalva maneuver [7]. The average volume of puffs has been reported to be 70 % larger (*p* < 0.001) and the duration of puffs about 60 % longer (*p* < 0.01 ) with a fourfold longer breath-holding time while smoking marijuana as compared to tobacco use [22]. This also resulted in inhalation of 2.8 to 3.3 times more insoluble particulates (tar) with deposition of 32 % to 35 % more particles compared to cigarette smoking, which may contribute to significant inflammation and epithelial injury [22].

## Marijuana and pulmonary infections

Alveolar macrophages play a significant role in the biological defense mechanisms of the lung via phagocytosis and destruction of pathogens. Therefore, impairment of this basic function is likely to increase the incidence of infections. Based on findings of rodent studies, Sherman et al. [23] designed a study to investigate the effects of smoking marijuana on human alveolar macrophages. They demonstrated that marijuana smoking decreased the ability of alveolar macrophages to destroy ingested Candida albicans, but it did not result in alteration of respiratory burst or phagocytosis. Baldwin et al. [24] provided further evidence of impairment in alveolar macrophage function due to cannabis use. Bronchoscopy and BAL were performed on 56 subjects: 40 males and 16 females; mean age: 34.4 ± 8.4 yr (including 22 non smokers, 10 marijuana smokers, 13 crack cocaine smokers, and 11 tobacco smokers). The alveolar macrophages were collected for analysis. Macrophages from marijuana smokers were impaired in their ability to phagocytose Staphylococcus aureus (*p* < 0.01), kill both bacteria and tumor cells (*p* <0.01), and produce cytokines.

However, clinical evidence of increased incidence of pulmonary infections as a result of marijuana use is less robust and largely anecdotal. A few case reports have documented occurrence of aspergillosis related to marijuana use in immunocompromised patients. Hamadeh et al. [25] reported a case of fatal pulmonary aspergillosis in a 34-year-old bone marrow transplant recipient with an extensive history of daily marijuana use prior to admission. Cescon et al. [26] also reported a similar case of invasive pulmonary aspergillosis in a patient with colorectal cancer who had been smoking marijuana for the treatment of chemotherapy-induced nausea six weeks prior to presentation. Case reports of marijuana-associated aspergillosis have been documented in patients with rheumatoid arthritis on oral corticosteroids as well as a history of Tetralogy of Fallot [27] .

In these case reports, the pulmonary infections have been attributed to the contamination of marijuana by fungi and to the fact that marijuana is smoked without a filter. This has been confirmed by studies performed by Verweij et al. [28] who cultured samples of tobacco and marijuana for mold. Marijuana was found to be heavily contaminated with both *Penicillium* species and *Aspergillus* fumigatus.

Marijuana inhalation techniques have also been linked to the transmission of tuberculosis. Munckhof et al. [29] described this phenomenon after identifying 149 contacts from five index cases of pulmonary tuberculosis in Australia. In these subjects, marijuana water pipe (bong) smoking was common among both cases and contacts. Therefore sharing a marijuana water pipe with an index case was identified as a risk factor for acquisition of tuberculosis infection in this subpopulation (OR 2.22, 95 % CI 0.96–5.17). Thu et al. [30] also evaluated three index cases of cavitary pulmonary disease and 34 identifiable positive contacts. A sixfold risk of transmission of tuberculosis was identified among contacts sharing a bong with an index case (odds ratio 6.5, confidence interval 1.4–30.4, *p* = 0.016).

“Hotboxing” is another method of recreational marijuana use reported to facilitate transmission of tuberculosis. This involves multiple persons smoking inside a closed car to repeatedly inhale exhaled smoke. Oeltmann and colleagues [31] published a case series of 11 index cases with 121 potentially exposed contacts (54 friends, 67 other contacts). Among those contacts who reported or were observed “hotboxing”, 11 (79 %) of 14 who received a Tuberculin Skin Test had a positive result. The association described between marijuana inhalation and tuberculosis transmission remains ambiguous, however, as it is not clear whether the increased risk is due to closer proximity to an actively infected TB case versus the result of impaired biological defense mechanisms due to marijuana use.

## Marijuana and lung cancer

A critical yet unresolved public health issue is whether cannabis smoking can increase the risk of lung cancer. Since marijuana smoke contains many of the same chemicals as tobacco smoke in addition to 60 cannabinoid compounds [32] it is plausible to raise the hypothesis that it may be carcinogenic. Several lines of evidence justify this concern: marijuana and tobacco smoke contain many of the same potent carcinogens and the condensate from marijuana smoke is even more toxic than that from tobacco smoke [32]. Furthermore, marijuana smoking results in greater delivery of tar to the lungs because it is associated with deeper inhalation and longer breath-holding times, and marijuana cigarettes are unfiltered [7, 22]. In addition, studies looking at bronchial biopsies have demonstrated that marijuana users show inflammation of the airways, but also histological and molecular changes indicative of precancerous changes, similar to those found in tobacco smokers [12, 13, 33]. Marijuana is also widely acknowledged to stimulate the mitogen-activated kinase (MAPK) pathway, which is a major stimulant for developmental and malignant cell growth. Exposure to cannabis was associated with an increased incidence of acute myelomonocytic leukemia (AMML) in the pediatric population after in-utero exposure to cannabis [34].

Even though there is molecular, cellular, and histopathological evidence for a potential carcinogenic effect of marijuana, the epidemiologic studies yielded inconsistent results.

In a retrospective cohort study, 64,855 examinees aged 15–49 years enrolled in the Kaiser Permanente health maintenance organization in northern California were followed for a mean length of 8.6 years [35]. Former and current users of marijuana failed to show an increased incidence of cancers at all sites after adjusting for tobacco use. Marijuana use was not associated with common tobacco-related cancers or with cancers of the lung, colorectal, melanoma, breast, prostate, and cervix. Among non-smokers of tobacco cigarettes, ever having used marijuana was associated with an increased risk of prostate cancer and a marginally increased risk of cervical cancer. However, the study was limited by the relatively young age of the subjects (average age was 48 years) and a follow-up period that was not long enough to allow for the development of cancer.

Also, a well designed population-based case–control study in Washington state failed to find any increased risk for the development of upper airway cancers in association with marijuana use (RR 0.9; 99 % CI 0.6–1.3) or any trend towards dose response in relation to the frequency and duration of use [36].

Another large population-based case–control study of 1,212 cancer cases of the upper aero-digestive tract in Los Angeles and 1,040 cancer-free controls also showed no positive association when adjusting for several confounders including nicotine use [37].

However, a case–control study conducted by Aldington et al. in New Zealand [38] included 79 cases of lung cancer and 324 controls. Subjects and controls were <55 years old. While marijuana smoking (lifetime use of more than 20 joints) was not associated with a significant increased risk of lung cancer, the subjects in the highest tertile of use (>10.5 joints/year with one joint/year equivalent to smoking 365 joints or filled pipes) had the highest risk RR 5.7 (95 % CI 1.5-21.6) after adjustment for confounders. Using logistic regression, the risk of lung cancer was estimated to increase by 8 % ( 95 % CI 2–15 %) for each joint/year of cannabis smoked after adjustment for confounding variables such as cigarette smoking, occupation, family history, etc. In this study, the association with lung cancer was found only in the third tertile, which contained a small number of patients (14 patients) and there was no dose-dependent relationship noted.

Furthermore, Callaghan et al. [39], in a population-based cohort study, examined 49,321 men aged 18–20 years old (military conscripts) in Sweden recruited between 1969–1979. The participants were tracked until 2009 (40 years) for incident lung cancer outcome using several Swedish patient registries. The study reported a twofold risk (hazard ratio 2.12, 95 % CI 1.08-4.14) of developing lung cancer over the 40-year follow-up period after statistical adjustment for baseline tobacco and alcohol use, co-existing respiratory conditions, and socio-economical status.

Because case control studies yielded mixed results, several authors presented pooled analyses of smaller studies. Berthiller et al. [40] presented three case–control studies conducted in Tunisia, Morocco, and Algeria, three areas of cannabis consumption and production. The study included 430 cases (all men) and 778 controls. All cannabis smokers were tobacco users. The study found that there was a 2.4-fold increase in the risk of lung cancer among ever cannabis smokers compared to never users after adjustment for age, tobacco smoking, and occupational exposure. The authors acknowledge the potential of residual confounding by tobacco smoking (tobacco and marijuana are habitually smoked together in that part of the world).

In Tunisia, in a hospital-based case–control study, the risk of lung cancer in prior cannabis users was studied in 149 new lung cancer cases and 188 controls [41]. The odds ratio for the past users of cannabis and lung cancer was 4.1 (95 % CI: 1.9-9.0) after adjustment for age, tobacco use, and occupational exposure. There was no clear dose–response relationship between the risk of lung cancer and the intensity or duration of cannabis use. Because in Tunisia many cannabis smokers mix cannabis with tobacco, tobacco may be a major confounder for these results.

Mehra et al. [42] published a systematic review of the impact of marijuana smoking on the development of premalignant lung changes and lung cancer. 19 studies entered into analysis from 186 initially identified. The authors found an association of marijuana smoking with increased tar exposure, alveolar macrophage tumoricidal dysfunction, increased oxidative stress, and bronchial mucosa histopathologic abnormalities compared to tobacco smokers and nonsmoking controls. The study did not find a significant association between marijuana use and lung cancer after adjusting for tobacco use. The authors raise the question of methodologic deficiencies in the studies included in the analysis that may have accounted for the results.

Zhang et al. [43] published a study which included 2,159 lung cancer cases and 2,985 controls pooled from six case-controlled studies within the International Lung Cancer Consortium. There was little evidence for an increased risk of lung cancer among habitual or long-term cannabis smokers. The authors however, mention that the possibility of potential adverse effects of heavy consumption could not be excluded.

The epidemiological evidence for an association between cannabis and lung cancer is limited and conflicting. Case series have suggested a causative role for cannabis for lung cancer in young adults. The case–control studies published to date have suggested both the presence and absence of an association, but these studies have been limited by the inability to quantify use, confounded by combined cannabis and tobacco or other substance use, or were undertaken in populations where cannabis use may have serious legal consequences resulting in potential reporting bias and poor response rates.

In addition, the interpretation of the epidemiological data is further limited by the design of these studies: case series represent uncontrolled observations from which causality cannot be inferred. Case–control studies, on the other hand, establish associations, but this again does not represent causation. In some of the studies in which a relationship between marijuana smoking and lung cancer was established, the association was weak and there was no dose–response relationship to strengthen the association.

Just as in the tobacco story, the long time lapse between smoking marijuana and the development of serious respiratory complications attenuates the individual’s perception of risk. As inconclusive as the epidemiological studies might be, prudence should warn individuals that marijuana smoking may result in serious short-term and long-term respiratory complications, potentially as drastic as those caused by tobacco smoke.

## Conclusions

The data presented, although not conclusive, suggest that in our practice, we should scrutinize marijuana smoking with the same diligence as we scrutinize tobacco use. The use of marijuana should be assessed and documented at each visit, both quantitatively and qualitatively, as precisely as possible, and patients should be provided with counseling using the same methods we use in treating tobacco addiction. The medical literature so far does not support a role for routine evaluation by pulmonary function tests or imaging studies, and until more definitive data is available, we do not recommend the regular use of these tests in the evaluation of habitual marijuana smokers.
